# Role of Pyroptosis and Ferroptosis in the Progression of Atherosclerotic Plaques

**DOI:** 10.3389/fcell.2022.811196

**Published:** 2022-02-03

**Authors:** Zhen Yang, Junhe Shi, Li Chen, Changgeng Fu, Dazhuo Shi, Hua Qu

**Affiliations:** ^1^ Xiyuan Hospital, China Academy of Chinese Medical Sciences, Beijing, China; ^2^ Cardiovascular Department, Peking University Traditional Chinese Medicine Clinical Medical School (Xiyuan), Beijing, China; ^3^ Academy of Integration of Chinese and Western Medicine, Peking University Health Science Center, Beijing, China; ^4^ National Clinical Research Center for Chinese Medicine Cardiology, Beijing, China; ^5^ NMPA Key Laboratory for Clinical Research and Evaluation of Traditional Chinese Medicine, Beijing, China

**Keywords:** ferroptosis, pyroptosis, macrophage, vascular endothelial cells, vascular smooth muscle cells, atherosclerotic plaque

## Abstract

Pyroptosis is a special way of programmed cell death which is dependent on the activation of cysteinyl aspartate specific proteinase 1 (Caspase-1) and Caspase-4/5/11. Ferroptosis is an iron-dependent cell death that characterized by the intra-cellular lipid peroxidation-mediated membrane damage. Pyroptosis or ferroptosis in macrophages, smooth muscle cells, and vascular endothelial cells are believed to be closely related to the progression of atherosclerotic plaques. Therefore, we discuss the role of pyroptosis and ferroptosis in the development of atherosclerotic plaques and may provide new strategies for the treatment of atherosclerosis.

## Introduction

Atherosclerosis (AS) is caused by multiple risk factors including hypertension, diabetes, obesity, smoking and high blood cholesterol level. The pathogenesis of AS has not yet been fully elucidated. Typical AS lesions refer to atherosclerotic plaques consisting of fibrous caps on the surface and a large number of lipid and necrotic cells that form the lipid core inside. AS plaque erosion and thrombosis after the rupture of AS plaque are the major causes of clinical cardiovascular events.

Recent studies show that pyroptosis and ferroptosis play important roles in the pathogenesis of AS. The levels of related molecules involved in pyroptosis, such as IL-18, IL-1β, Caspase-1, apoptosis-associated speck-like protein containing CARD (ASC), nucleotide-binding oligomerization domain, leucine- rich repeat and pyrin domain-containing 3 (NLRP3), are higher in unstable plaques compared to those in stable plaques (Shi et al., 2015). Atherosclerotic plaques contain a large number of foaming macrophages. In the course of plaque formation, cholesterol is phagocytosed by macrophages, forming foam cells and promoting macrophages to secrete numerous inflammatory cytokines, such as tumor necrosis factor-α (TNF-α), interleukin-6 (IL-6), IL-2. These cytokines aggravate local inflammatory responses and further promote macrophage pyroptosis, subsequently lead to further damage and rupture of plaques (Karunakaran et al., 2015; Ouimet et al., 2016). The formation of vulnerable atherosclerotic plaques is not only affected by macrophage foaming changes but also strongly associated with local active inflammation. In the case of substantial lipid aggregation, these lipids that cannot be cleaned in time induce the activation of pyroptosis signaling pathways, followed by the release of numerous inflammatory cytokines which promote the formation of locally vulnerable plaques.

Martinet et al. (2019) found that the formation of atherosclerotic plaques is closely related to the change of iron levels in the body. Iron overload can promote oxidative stress, lipid peroxidation and other pathological processes, leading to the instability of plaques. Lipid peroxidation, plaque hemorrhage and iron deposition are important features of advanced atherosclerotic plaques. This suggests that ferroptosis may be involved in the development of atherosclerotic plaques. Feng and Stockwell. (2018) found that inhibiting the activity of glutathione peroxidase 4 (GPX4), a key enzyme in ferroptosis, may lead to the accumulation of substantial lipid peroxides, thereby resulting in ferroptosis and the development of atherosclerotic plaques. The use of iron chelating agents can effectively reduce the surface volume of atherosclerotic plaques, stabilize plaques, and slow down the development of atherosclerotic plaques (Sung et al., 2012).

Therefore, it is necessary to discuss the role of pyroptosis and ferroptosis in the progression of atherosclerotic plaques.

## The Role of Pyroptosis in the Progression of Atherosclerotic Plaques

The pathological process of AS is characterized by endothelial disorders, low-density lipoprotein oxidation, monocyte and lymphocyte recruitment, and pro-inflammatory cytokine activation, resulted in migration and proliferation of vascular smooth muscle cells (VSMCs), foam cell formation, and cell death (Khatana et al., 2020). Pyroptosis of endothelial cells (ECs), VSMCs, and macrophages is strongly associated with AS and may be a novel target for the treatment of AS (Yu et al., 2020).

### Macrophage Pyroptosis Increases Atherosclerotic Plaque Vulnerability

At the early stage of AS, lipoprotein is deposited in the subendothelium. P-selectin, E-selectin, intercellular adhesion molecule 1 (ICAM-1) and vascular cell adhesion molecule 1 (VCAM-1) will be released and subsequently recruit monocytes to the atherosclerotic lesion site. Then the recruited monocytes will differentiate into macrophages after inflammatory stimulation (Chawla et al., 2011). Macrophages uptake modified lipoproteins, such as oxidative low-density lipoprotein (ox-LDL), and turn into cholesterol-rich foam cells, which will trigger a series of inflammatory responses that promote plaque formation (Libby, 2002). Macrophages, the major immune cells in AS, play an important role in both early AS plaque formation and late plaque rupture. Shi et al. (2017) found that in early AS, moderate macrophage death alleviate the inflammatory response and reduced the matrix metalloproteinase (MMP) synthesis. However, in late stage of AS, excessive cell death and insufficient efferocytosis enlarge the plaque necrotic lipid core, enhance the inflammatory response, and increase the plaque vulnerability. Other than that, accumulating evidences from recent studies suggest that macrophage pyroptosis plays an important role in the formation and rupture of AS vulnerable plaques (Xu et al., 2018).

The role of NLRP3 inflammasome activation in macrophages is particularly critical in mediating macrophage pyroptosis, releasing inflammatory cytokines, and promoting AS development. In the AS lesions, the NLRP3 inflammasome can be activated by substances like ox-LDL and cholesterol crystallization, followed by subsequent transformation of Caspase-1 precursors into bioactive Caspase-1, causing macrophage pyroptosis and enabling the extracellular release of pro-inflammatory cytokines IL-18 and IL-1β (Jia et al., 2019). Classical macrophage pyroptosis depends on Caspase-1 activation. Inflammasomes can regulate Caspase-1 activation, which in turn promotes the maturation of inflammatory factor precursors pro-IL-18 and pro-IL-1β. Stimulated by various pro-inflammatory factors, macrophages can secrete numerous inflammatory factors (such as IL-6, IL-1β, TNF-α, etc.), chemokines (such as IL-8, MCP-1, CXCL1 etc.) and other cytokines (such as MMP-1, MMP-8, MMP-9, M-CSF etc.), leading to lipid accumulation and matrix degradation, and result in AS plaque rupture (van der Heijden et al., 2017).

Regulation of macrophage pyroptosis might be a promising approach to enhance plaque stability and delay AS progression. Recently, small molecule inhibitors targeting the NLRP3 inflammasome have become a hot topic and are expected to be a new target for AS treatment. MCC950 has been identified as the most representative small molecule NLRP3 inhibitor. It can impede macrophage pyroptosis by inhibiting the conversion of macrophage to foam cells through suppressing ox-LDL uptake and increasing cholesterol efflux, which increased plaque stability in ApoE^−/−^ mice (van der Heijden et al., 2017). Therefore, regulating NLRP3 stimulators, suppressing NLRP3 inflammasome activation, and inhibiting macrophage pyroptosis are critical in controlling AS immuno-inflammatory response and delaying AS progression.

### Vascular Endothelial Cells Pyroptosis Increases Atherosclerotic Plaque Lipid Deposition

VECs are monolayer cells lining on the surface of blood vessels, and VECs dysfunction is responsible for many pathological changes in the formation and development of AS (Gimbrone and García-Cardeña, 2016). The damage of VECs occurs in the starting stage of AS, which is also a key stage. Recently, it has been reported that multiple pathological risks during AS can lead to VECs pyroptosis. For example, oxidized lipid components activated by Caspase-1 through reactive oxygen species (ROS) can induce VECs pyroptosis (Yin et al., 2015). High homocysteine can induce VECs pyroptosis by activating the Caspase-1-dependent inflammasome, leading to endothelial dysfunction (Xi et al., 2016). Ox-LDL can induce VECs pyroptosis through the miR-125a-5p/TET methylcytosine dioxygenase 2 (TET2) signaling pathway (Zhaolin et al., 2019). Long-chain non-coding RNA (lncRNA) metastasis-associated lung adenocarcinoma transcript 1 (MALAT1) can competitively bind microRNA-22, activate the NLRP3 inflammasome, and promote high glucose-induced VECs pyroptosis (Song et al., 2019).

Recent studies revealed that some risk factors can induce AS by activating NLRP3 inflammasome in ECs (Grebe et al., 2018). Koshiyama et al. (2018) found that VECs exposed to cadmium can lead to the activation of NLRP3. The NLRP3 pathway not only activates ECs to enhance monocytes, but also leads to the death of ECs. The activation of NLRP3 destroys the integrity of the vascular endothelium, causes the local lipid deposition, and promotes the formation of atherosclerotic plaques. It has also been reported that NLRP3 inflammasome can cause pyroptosis in arterial plaques, which is closely related to plaque rupture and vascular inflammation (Hoseini et al., 2018). Thus, the activation of NLRP3 and its mediated ECs pyroptosis may play crucial parts in the formation and development of atherosclerotic plaques. Caspase-1, as a downstream factor of NLRP3, can also activate ECs mediated by IL-18 and IL-1β (Motekallemi et al., 2019). Wu et al. (2018) found that nicotine produced by smoking activates the NLRP3 inflammasome, promotes VECs pyroptosis and IL-lβ, IL-18 release, and accelerates AS progression. Shimamura et al. (2018) found that excessive cholesterol and oxide lipid in ApoE−/− mice can induce ECs pyroptosis by activating Caspase-1. However, the inhibitiors of NLRP3 can inhibit the pyroptosis of VECs, such as dihydromyricetin and biochanin, which have been reported to achieve the inhibition of NLRP3 by activating the red system-derived nuclear transcription-related factor 2 (Nrf2) pathway (Kim et al., 2010).

In addition, several studies indicated that non-coding ribonucleic acid (ncRNA) is involved in regulating ECs pyroptosis of AS. For example, Song et al. (2019) demonstrated that lncRNA MALAT1 plays a role in human ECs treated with high glucose for upregulation of EA.hy926 cells. LncRNA MALAT1 promotes pyroptosis by upregulating NLRP3. LncRNA MALAT1 knockdown significantly inhibits high glucose-induced ECs pyroptosis. Research on the mechanism of lncRNA MALAT1 promoting pyroptosis has found that miR-22 is a target of lncRNA MALAT1 and there is a negative correlation between miR-22 and lncRNA MALAT1. Overexpression of miR-22 can inhibit lncRNA MALAT1 in ECs. LncRNA MALAT1 competes with miR-22 to affect NLRP3 expression, thereby promoting hyperglycemia-induced VECs pyroptosis. Wang et al. (2020) found that miR-103 protects coronary ECs from H_2_O_2_-induced oxidative stress injury through the BCL2 Interacting Protein 3 (BNIP3)-mediated late autophagy and anti-pyroptosis pathways. MiR-125a-5p inhibits the post-transcriptional expression of TET2, leading to activated nuclear factor κB (NFκB), increased ROS production, mitochondrial dysfunction, and aberrant deoxyribonucleic acid (DNA) methylation, following the activation of NLRP3 and Caspase-1 and eventually results in the pyroptosis of VECs (Zhaolin et al., 2019). Wang et al. (2019) indicated that miR-125A-5p inhibits VECs pyroptosis by downregulating chemokine 4-like (CCL4) and inhibiting the expression of IL-1β, Caspase-1, ASC and NLRP3 (Li et al., 2018). Increased level of forkhead box O3 (FOXO3) in VECs by miR-30c-5p can lead to the inhibition of NLRP3-mediated pyroptosis. These results suggest that ncRNA plays different roles in regulating pyroptosis in different cells (Li et al., 2018).

VECs pyroptosis can lead to an enhanced inflammatory response in the vascular wall and the destruction of the vascular membrane. During pyroptosis, the massive secretion of cytokines, such as VCAM-1, ICAM-l, P-selectin, IL-18, and IL-1β, can trigger the adhesion of monocytes or macrophages to VECs, further aggravating the vascular wall inflammation and the degree of AS lesions (Mestas and Ley, 2008). Furthermore, the activation of Caspase-1 promotes the expression of mouse CXC-chemokine ligand 16 (CXCL16) and its CXC-chemokine receptor 6 (CXCR6), which stimulates the migration of T lymphocytes to the arterial intima and the inflammatory response in the vascular wall (Sheikine and Sirsjö, 2008). Pyroptosis reduces the number of VECs and disrupts endothelial integrity, leading to increased endothelial permeability, which promotes VSMCs migration and deposition to the inner membrane. Lopez-Pastrana et al. (2015) showed that inhibiting Caspase-1 activation and reducing VECs pyroptosis can promote angiogenesis. Zhang et al. (2018) suggested that melatonin inhibits VECs pyroptosis via the maternally expressed gene 3 (MEG3)/miR-223/NLRP3 signaling axis, and its alternative is expected to provide a new approach for AS control. MicroRNA-30c-5p inhibits NLRP3 inflammasome-mediated VECs pyroptosis by enhancing the expression of FOXO3 and is expected to provide new ways for AS control (Li et al., 2018).

### Vascular Smooth Muscle Cells Pyroptosis Enlarges Atherosclerotic Plaque Necrotic Core

VSMC and the extracellular matrix produced by it, such as collagen and elastin, are the major components of atherosclerotic plaque fibrous caps. Thus, VSMCs are closely related to plaque stability. In early stage of AS, activated VSMCs have a strong proliferative and migratory capacity to migrate from the middle membrane to the inner membrane, and have an important protective effect against plaque rupture by secreting the extracellular matrix and stabilizing the fibrous caps. However, in late stage of AS, VSMCs are activated under cholesterol load due to substantial lipid accumulation within the plaque, leading to the phenotypic transformation of VSMCs to macrophage-like cells that can promote the atherosclerotic inflammatory response and enlargement of the plaque necrotic core (Bennett et al., 2016). VSMCs pyroptosis plays an important role in promoting the progression of AS, leading to thinning of the plaque fibrous caps, enlargement of the necrotic core, and infiltration of macrophages, which all contribute to vulnerable plaques. VSMCs pyroptosis, on the one hand, increases the instability of plaques, and on the other hand, releases a large number of pro-inflammatory factors that cause persistent inflammation and destroy the structure of blood vessel walls.

**FIGURE 1:**
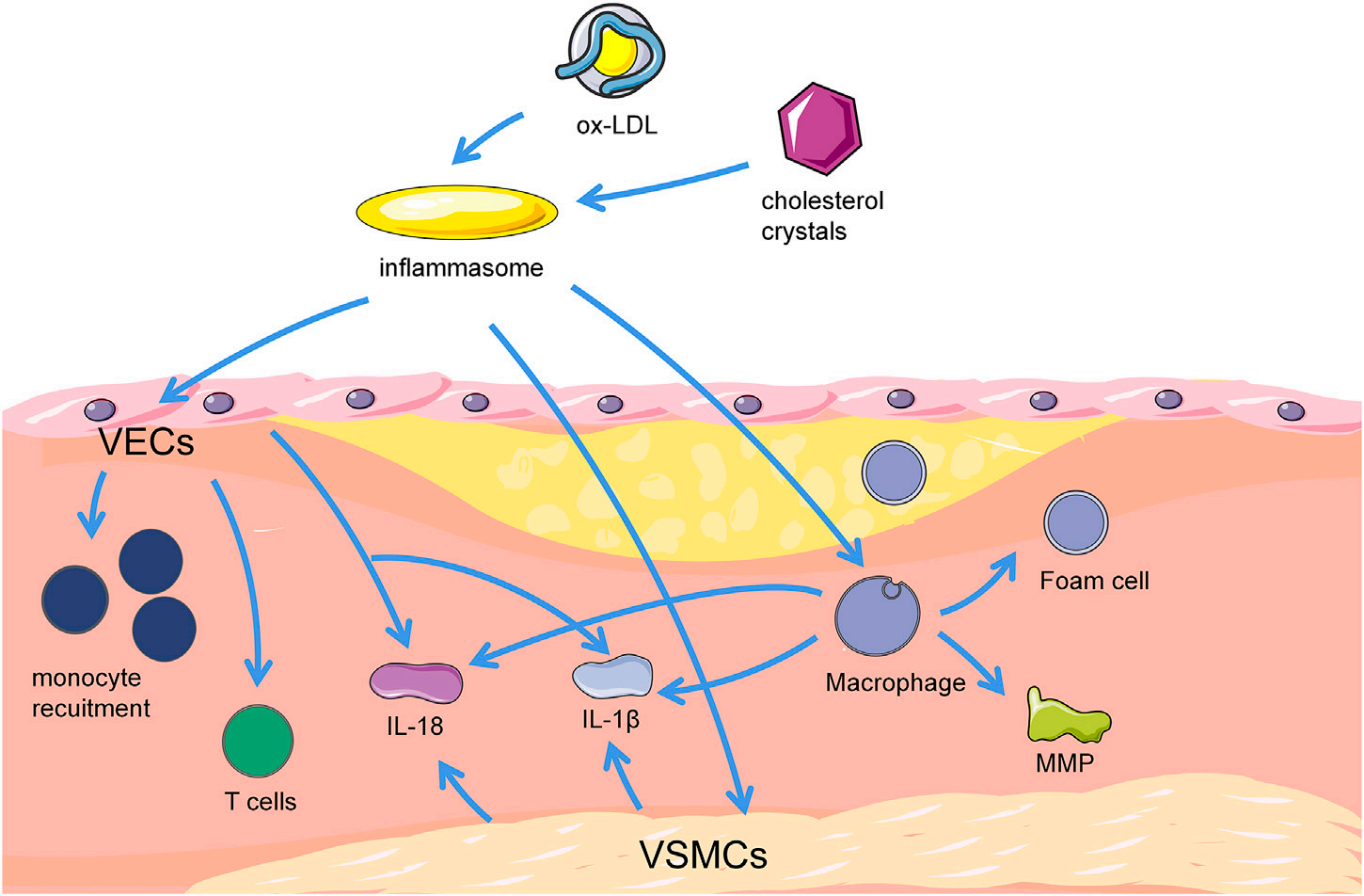
Correlation between pyroptosis and progression of atherosclerotic plaques. Cholesterol crystal, and ox-LDL can activate inflammasomes in ECs, macrophages and VSMCs, trigger cellular pyroptosis and stimulate AS. Pyroptotic ECs can mediate T cell migration to the inner membrane and promote monocyte recruitment. Furthermore, pyroptotic ECs release proinflammatory cytokines, such as IL-18 and IL-1β, and result in vascular inflammation. Macrophages uptake cholesterol crystals and turn into lipid containing foam cells subsequently. Macrophage pyroptosis mediate the production of pro-inflammatory mediators and cytokines, including MMP, IL-18, and IL-1β. These pro-inflammatory mediators and cytokines also trigger foam cell pyroptosis, thereby forming the necrotic core of plaques in advanced lesions. Pyroptotic VSMCs release pro-inflammatory cytokines like IL-18 and IL-1β, and attenuate stability of fibrous caps via loss of collagen and matrix, causing inflammation, promoting plaque instability or erosion, and potentially worse AS. VSMCs, vascular smooth muscle cells. VECs, vascular endothelial cells. MMP, matrix metalloproteinase. IL-18, interleukin-18. IL-1β, interleukin-1β.

Absent in melanoma 2 (AIM2) pattern recognition receptors can activate Caspase-1 via the classical cellular pyroptosis pathway and subsequently mediate cellular pyroptosis by cleavage of GSDMD (Man et al., 2015). Recent studies showed that overexpression of AIM2 promotes VSMCs migration in the aortic tissue of ApoE^−/−^ mice, leading to increased atherosclerotic plaque area. *In vitro* experiments found that ox-LDL can time-dependently increase AIM2 in VSMCs and thus speculated that ox-LDL can mediate VSMCs pyroptosis by AIM2 to promote AS lesions (Pan et al., 2018). VSMCs that undergo pyroptosis can also release inflammatory factors such as IL-1β and IL-18, exacerbate the inflammatory response, reduce collagen and extracellular matrix synthesis, weaken the stability of fibrous caps, and increase plaque vulnerability, thus lead to plaque erosion and rupture. The Caspase-1 inhibitor VX-765 inhibits the pyroptosis of smooth muscle cells, and the substance can also inhibit foam cell formaton by inhibiting IL-1β (He et al., 2021). It is also expected to provide a new insight for AS control (Figure 1).

## The Role of Ferroptosis in the Progression of Atherosclerotic Plaques

A human epidemiological study shows that serum ferrin levels and transferrin saturation are positively correlated with the incidence of myocardial infarction and peripheral vascular disease (Holay et al., 2012). It can be seen that the content of iron in the peripheral blood of the body may be associated with the occurrence and development of vascular disease. Bai et al. (2020) indicated that Fer-1 can effectively inhibit the formation of AS in ApoE gene knockout mice induced by a high-fat diet. Compared with control mice, iron accumulation and lipid peroxidation levels were partially inhibited in Fer-1 added mice, with an upregulation in the expression of SLC7A11 and GPX4, which are protective factors of ferroptosis. Again, cell experiments *in vitro* verified the results of animal experiments, showing that Fer-1- could reduce endothelial cell ferroptosis and improve ECs viability by downregulating the expression of pro-inflammatory factors. These findings indicate that ferroptosis plays a significant role in the development of atherosclerotic plaques by reducing lipid peroxidation and protecting ECs function. Studies have shown that ferroptosis will be triggred in macrophages, VSMCs, and VECs under certain pathological conditions (Sampilvanjil et al., 2020). Therefore, removing excess iron and reducing ROS production may be new strategies for future treatment and prevention of AS.

### Foamy Macrophage Ferroptosis Stimulates Angiogenesis and Aggravates Plaque Instability

Macrophages are involved in maintaining the homeostasis of iron levels in the body. Malhotra et al. (2019) found a reduction of macrophage pro-inflammatory markers and AS in hepcidin knockout LDLR^−/−^ mice. In inflammatory environment of AS, the relative homeostasis between macrophage cholesterol phagocytosis and excretion is broken, and M1-type macrophages form foam cells after scavenger receptors phagocytose ox-LDL, thus promoting plaque production. In atherosclerotic plaques, the oxidation and phagocytosis of low density lipoprotein by macrophages as well as the formation of foam cells play a vital role in the development of plaques. Foamy macrophages undergo ferroptosis and release cellular components and lipids to form the necrotic core, which is one of the key pathological progress for AS. A large amount of ox-LDL is phagocytosed by macrophages to form foam cells, sequentially deposited in the endothelium of blood vessels. Foam cells upregulate proteolytic enzymes such as MMP and degrade extracellular matrix structures, thereby leading to the rupture of atherosclerotic plaques. Studies have found that there are two types of macrophages in atherosclerotic plaques (Bouhlel et al., 2007), of which M1-type macrophages have low expression of ferroportin, leading to the promotion of AS. When M1-type macrophages ingest a large amount of ox-LDL to form foam cells, a large number of foam cells gather into lipid fibrous caps and induce the release of inflammatory factors such as MMP-1, MMP-3 and MMP-9. Meanwhile, the paracrine effect also induces the proliferation of VSMCs-derived foam cells and their migration to the arterial intima (Ley et al., 2011). This accelerates the rupture of unstable atherosclerotic plaques and promotes the occurrence of acute cardiovascular adverse events.

During atherosclerotic plaque formation, oxygen consumption increases in the vascular wall, and the plaque lesion occurs hypoxia. Hypoxia stimulates angiogenesis, which is highly permeable and brittle. The highly inflammatory environment of advanced plaques is more vulnerable to disrupting the fragile microvasculature, leading to intraplaque hemorrhage (IPH). Ferroptosis has an impact on macrophages of IPH, subsequently leading to increased plaque instability and necrotic core expansion. Macrophages in the IPH region are distinct from M1 and M2 macrophages, which are characterized by lack of lipid deposition, low expression of pro-inflammatory cytokines, and high expression of CD163. As a result, they are named M(Hb) macrophages that have lower iron levels. Iron level is indirectly related to the activation of hypoxia-inducible factor-1α (HIF-1α). Iron ions are an important cofactor in the hydroxylation process of HIF-1α by prolyl hydroxylases (PHDs). Abnormal accumulation of iron enhances the activity of PHDs and accelerated degradation of HIF-1α. However, the removal of iron ions with ferroptosis inhibitor, deferoxamine (DFO), inhibited the PHDs activity and induced the expression of HIF-1α (Renassia and Peyssonnaux., 2019). Activated HIF-1α can induce the transcription of pro-angiogenic genes such as vascular endothelial growth factor (VEGF) (Cornelissen et al., 2019). In angiogenesis experiments, the treatment of ECs with cultured supernatants of M(Hb) macrophages resulted in more angiogenesis, greater vascular permeability, and activation of NFκB (Guo et al., 2018). Therefore, M(Hb) macrophage ferroptosis inhibits the function of iron-dependent PHDs, ultimately promoting VEGF-mediated intraplaque angiogenesis, increased vascular permeability, recruitment of inflammatory cells, and increased plaque instability.

### Ferroptosis of Vascular Endothelial Cells Increases Atherosclerotic Plaque Lipid Deposition

VECs are the barrier to protect the internal surface of blood vessels. Upon impairment of VECs, the activation of inflammatory factors and the deposition of oxidized lipids under intima can accelerate the formation of vascular endothelial plaques (Cancel et al., 2016). It has been found that excess iron aggravates the cellular oxidative stress levels by producing massive ROS through Fenton responses, with overexpression of inflammatory factors such as VCAM-1, monocyte chemoattractant protein-1 (MCP-1) and ICAM-1, thus promoting endothelial injury and accelerating lipid deposition (Marques et al., 2019; Vinchi et al., 2020). Ox-LDL can induce VECs damage to promote AS, which is associated with ferroptosis. An *in-vivo* study showed that mice treated with ox-LDL or erastin can damage mitochondria of aortic ECs and subsequently increase the levels of ROS, lipid peroxidation, and malondialdehyde (MDA). However, Fer-1 inhibited the generation of these peroxidation products, suggesting that ox-LDL can induce VECs ferroptosis (Li et al., 2020). The results showed that iron overload causes reduced vascular NO bioavailability in ApoE^−/−^ mice, and long-term iron injection into mice reduces the number of antioxidants such as peroxidase and superoxide dismutase in the blood and liver, thereby causing ECs damage. Chronic iron overload exacerbates the progression of AS through ROS pathways in ApoE^−/−^ mice, possibly due to diastolic and contractile factor imbalance in impaired VECs synthesis (Marques et al., 2019). Whereas this process can be reversed by Fer-1 treatment (Xiao et al., 2019). Furthermore, there is direct evidence that VECs ferroptosis promotes AS by accelerating endothelial dysfunction during ROS-mediated lipid peroxidation. Ferroptosis inhibitors improve ox-LDL-induced ECs damage and lipid peroxidation (Bai et al., 2020). Fer-1 and iron chelator mesylate can exert anti-ferroptosis, rescue ferroptosis damage in ECs, and restore antioxidant activity and iron metabolism (Wang and Tang, 2019).

### Ferroptosis Induces Vascular Smooth Muscle Cells Proliferation and Calcification

**FIGURE 2:**
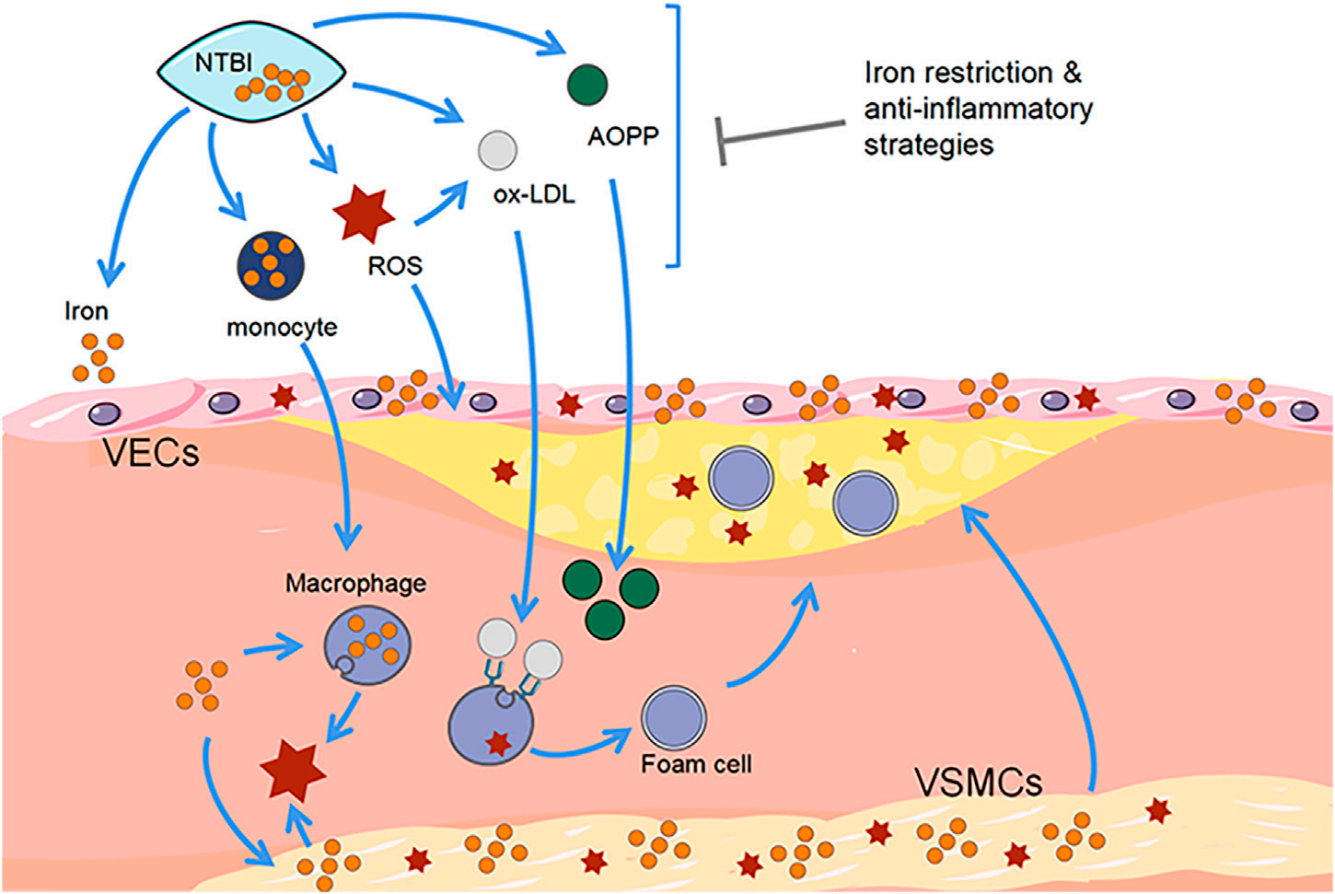
Correlation between ferroptosis and progression of atherosclerotic plaques. Free iron promotes the development of AS and increases the risk of cardiovascular disease. The detrimental effects of iron accumulation resulting from the presence of macrophages, endothelial cells, and vascular smooth muscle cells exacerbate the formation of atherosclerotic plaques. Disease progression can be prevented by iron-consuming strategies. VSMCs, vascular smooth muscle cells. VECs, vascular endothelial cells. NTBI, non-transferrin-bound iron. ROS, reactive oxygen species. Ox-LDL, oxidized low density lipoprotein. AOPP, advanced oxidation protein products.

VSMCs are located in the vascular middle membrane with the role of regulating vascular tension and maintaining vascular elasticity. VSMCs can switch from a contractile phenotype into a synthetic phenotype, causing vascular sclerosis and lumen stenosis, leading to the occurrence of AS (Numaga-Tomita et al., 2019). Iron overload can cause dysfunction in VSMCs proliferation, apoptosis, ROS production, phenotypic conversion, and calcification (Malhotra et al., 2019). Animal experiments show that iron mainly deposited in the arterial membrane, allowing VSMCs in the plaque to obtain a macrophage-like phenotype, resulting in migration of iron overloaded VSMCs, reduced collagen and increased foam cells and lipid levels, forming unstable plaques and easy to rupture. While iron accumulation of VSMCs also creates pro-oxidative microenvironment and promotes the development of foam cells and plaque rupture (Guo et al., 2017). Vinchi et al. (2020) found that treating VSMCs with ammonium ferric citrate can increase levels of inflammation and apoptosis as well as promote monocyte adhesion and migration. At the same time, iron overload within VSMC stimulates its proliferation, causes ROS generation, and aggravates the pro-oxidative microenvironment, which is more conducive to foam cell formation and increases plaque instability (Ramakrishnan et al., 2018). In addition, M1-type macrophages produce platelet-derived growth factor or transformed growth factor-β, which can cause the proliferation of VSMCs and induce their migration to the vascular intima, thus driving the development of AS. This suggests that iron levels within macrophages can indirectly affect VSMCs activation. Iron can also regulate the endoplasmic reticulum stress (ERS)-related pathways. Wang et al. (2017) found that by increasing the iron content in VSMCs, ERS and proliferation of VSMCs can be reduced, while reduced ERS can alleviate the process of AS. Furthermore, iron can also induce the calcification of VSMCs through the IL-24 and bone morphogenetic protein-2 (BMP2) pathways (Kawada et al., 2018). In conclusion, it can be speculated that excessive iron levels and stimulation of various factors induce VSMCs ferroptosis, leading to abnormal proliferation and calcification of VSMCs, and eventually cause vascular lumen stenosis, thus promoting the progression of atherosclerotic plaques (Figure 2).

## Conclusion and Perspectives

Pyroptosis is a unique form of cell death characterized by membrane perforation, cell swelling, membrane rupture and release of cell content. Caspase-1, Caspase-4, Caspase-5, Caspase-11 are involved and activated by specific inflammatory factors, such as double-stranded deoxyribonucleic acid (dsDNA) and invading pathogens during the pyroptosis process. There is a growing number of evidences indicating that the activation of NLRP3 inflammasome is a key regulatory factor and maybe a biomarker of pyroptosis in the development of atherosclerotic plaques. Pyroptosis has been found in macrophages, VECs, and VSMCs. Some ncRNAs or other molecules are involved in regulating the pyroptosis of VECs. Although the role of pyroptosis in AS has been already studied extensively, its specific molecular mechanism remains unclear. Further studies are needed to investigate the regulatory role of ncRNA in AS pyroptosis, determine the non-classical inflammasome pathway and the function of GSDMD in AS. Elucidating the molecular mechanism of pyroptosis will help us to understand what role it plays in the development of AS, which is crucial for developing anti-AS drugs that target the inflammasome signaling pathway.

The function of ECs is damaged when the lipid metabolism in the body circulation is disturbed. Macrophages in the blood circulation mediate the oxidative modification of infiltrated LDL beneath VECs to form ox-LDL, while recruited macrophages and VSMCs engulf ox-LDL and forming foam cells, finally, ox-LDL deposit abnormally in the subcutaneous artery to form atherosclerotic plaques. As stated above, many studies have been demonstrated that iron overload can alter macrophage function, such as promoting ROS production, inflammatory responses, foam cell formation, glycolysis and macrophage polarization. In addition, iron overload can also lead to VECs dysfunction, destroy VSMCs, stimulate immune cell aggregation, and accelerate the progression of AS. So the effect of iron on AS needs to be considered on the diagnosis and treatment of AS. However, when iron levels in the peripheral circulation are abnormal, whether ferroptosis directly occurs in macrophages, VSMCs, and VECs is still unclear. Therefore, to figure out what role ferroptosis plays in the occurrence and development of atherosclerotic plaques that cause clinical adverse cardiovascular events remains to be further explored in subsequent studies. In conclusion, ferroptosis has broad research potential as the role it plays in AS which will bring new strategies for the diagnosis and treatment of vascular diseases with further exploration (Table 1).

**TABLE 1:** Comparison and summary of the effects of pyroptosis and ferroptosis on different cells.

	Pyroptosis	Ferroptosis
Macrophages	Increases atherosclerotic plaque vulnerability	Stimulates angiogenesis and aggravates plaque instability
VECs	Increases atherosclerotic plaque lipid deposition	Increases atherosclerotic plaque lipid deposition
VSMCs	Enlarges atherosclerotic plaque necrotic Core	Induces proliferation and calcification

VSMCs, vascular smooth muscle cells. VECs, vascular endothelial cells.
